# Editorial for Special Issue on Biosensors for Biomedical and Environmental Applications

**DOI:** 10.3390/mi15050607

**Published:** 2024-04-30

**Authors:** Antonella Battisti

**Affiliations:** CNR—Nanoscience Institute, p.zza San Silvestro 12, 56127 Pisa, Italy; antonella.battisti@nano.cnr.it

A sensor is typically defined as a device able to transform a physical quantity of interest into a different kind of signal that can be easily measured and recorded. Notably, every living organism is provided with biological sensors that, just like artificial sensors, are able to detect quantities like physical (light, temperature, movement, …), metabolic (oxygen levels, glucose levels, …), and biochemical (interactions with hormones, antigens, cytokines, …) stimuli. The International Union of Pure and Applied Chemistry (IUPAC) in its Glossary, commonly referred to as the “Gold Book”, defined a biosensor as “a device that uses specific biochemical reactions mediated by isolated enzymes, immunosystems, tissues, organelles or whole cells to detect chemical compounds usually by electrical, thermal or optical signals” [1]. Strictly speaking, then, the term “biosensor” specifically refers to devices relying on a biological component as the actual sensing element. This component can be a biomolecule, a whole cell, a tissue fragment, or a microorganism [2,3,4,5,6,7]. However, commonly speaking, the same term is often used to define artificial systems used in the detection of biocomponents (biomolecules, microorganisms, biomarkers, …). The Special Issue “Biosensors for Biomedical and Environmental Applications” [8] covers the broader interpretation of the term, since it contains works concerning both biosensors narrowly defined and sensors for biocomponents.

Sensors incorporate not only the sensing element (or elements, in case of multiplexing devices) but also a substrate for the immobilization of the probe and a transducer that converts the triggering event into a detectable physical signal (optical, electrical, acoustic, mechanical, …). A suitable platform assembles these items together, and many efforts are being made to make such platforms easier to operate, smaller, and portable [9,10]. With a boost from the recent pandemic events, the fabrication of point-of-care systems turned out to be a new trend in the healthcare industry due to their ease of use, response rapidity, and low cost [11], and the same approach can also be found in the environmental sector. Improvements concerning any of the single components (sensing element, substrate, transducer, or platform assembly) can lead to more efficient, cheaper, and miniaturized devices able to assess the presence of multiple analytes in a single measure and to speed up the diagnostic pathway.

In a way, the first paper of the Special Issue “Biosensors for Biomedical and Environmental Applications” perfectly matches the IUPAC definition of biosensors. In the work by Martinović et al., the physiological state of Pen Shell Pinna Nobilis was evaluated by heart rate recording under potentially stressing conditions (https://doi.org/10.3390/MI13091549). Turning the perspective around, the living organism becomes the health indicator for the surrounding environment, e.g., a sensor for sea water pollution or parasites.

The paper by Olariu et al. reveals how HT-29 colorectal cancer cells can be distinguished from healthy blood cells in a low-conductivity medium by electromanipulation combining dielectrophoresis and electrical impedance spectroscopy (https://doi.org/10.3390/MI13111833). This methodology assesses the possibility of separating normal from cancer cells on the basis of their different dielectric properties. From the opposite point of view, this is also a case where a formerly characterized living cell could return information, e.g., about the surrounding electric field.

Similarly, oxidative stress due to an imbalance between the production of reactive oxygen species (ROS) and antioxidant defense systems can be detected by monitoring H_2_O_2_ levels in cell cultures media. Patella et al. demonstrate the effect of environmental variables such as different culture media and the presence of interfering species at different temperatures on an electrochemical sensor for effective oxidative stress quantification (https://doi.org/10.3390/mi13101762). Given the aforementioned considerations, in this case, the detection of H_2_O_2_ could also be used to obtain information from cells about the health state of the involved tissue, making the cells the sensing element of a biosensor.

Among the conventional biological sensing elements, enzymes and antibodies definitely play a major role. Enzymes are applied in the quantification of glucose, which is a crucial analyte, e.g., for the health monitoring of diabetic patients. Sigolaeva et al. propose to modify the surface of graphite-based electrodes for amperometry using a solution of a microgel–enzyme complex. The optimized coating allows the chosen enzyme, glucose oxidase, to have high sensitivity and reproducibility in the detection of β-D-glucose (https://doi.org/10.3390/MI14081629). Guan et al. also disclose a novel point-of-care device based on the immobilization of glucose oxidase on the surface of a copper electrode using a multi-material multi-layered film. This device shows promise in terms of performance and patient compliance since it can efficiently quantify the glucose levels in sweat samples (https://doi.org/10.3390/MI13122142). Antibodies are also commonly exploited to detect a plethora of biomarkers. In the paper by Montero-Arevalo et al., the electrochemical performances of bare gold and hexagonal crystalline silicon carbide electrodes were compared upon functionalization with the antibody Aβ1-28 for the detection of the amyloid-beta peptide (Aβ1-42), a reliable biomarker for Alzheimer’s disease (https://doi.org/10.3390/MI14061262).

Concerning intracellular measurements based on chemical fluorescent probes, fluorescein and its derivatives have always been a top choice for ratiometric imaging. Surzhikova et al. carried out a study to elucidate how the presence of viscogenic agents and temperature alterations can affect fluorescein’s ratiometric response. Their results enable the extraction of additional information from ratiometric data during fluorescein probing in biological samples as well as in other contexts such as biochemistry or microfluidics (https://doi.org/10.3390/MI14071442).

Environmental sensors are also considered, especially in the light of the Internet Of Things (IoT) technology. Kim et al. fabricated a porous PDMS sponge sensor with an embedded liquid metal and silver nanowires coating. The cost-effective, lightweight, and highly flexible porous structure shows excellent potential for use as part of strain or pressure sensors in wearable devices, robotic fingers, and healthcare instruments; however, it could also find possible application in mechanical sensors for environmental or industrial applications (https://doi.org/10.3390/MI13111998). The work by Wu et al. (https://doi.org/10.3390/MI14071395) aims to develop an in situ real-time data acquisition system for monitoring temperature and moisture in soils for smart agriculture applications. Soil parameters are collected by a wireless sensor connected to a cloud platform, and using a mobile phone application the operator can access predictions obtained on the basis of the collected data through machine learning algorithms. Similarly, Eldeeb et al. (https://doi.org/10.3390/MI14071314) propose a soil nitrate sensor capable of the real-time monitoring of nitrate levels in non-pretreated soils, giving immediate information about the soil’s health, an essential notion for sustainable agriculture.

Some innovative technological devices are also proposed in this Special Issue, including a high-throughput dispenser for viscous solutions to be used in the manufacturing of three-dimensional scaffolds for disease modeling (https://doi.org/10.3390/MI13101730), a multisensory system for an integrated quality assessment during the drone delivery of blood and blood components (https://doi.org/10.3390/MI13101664), and a microfluidic device for the detection of variations in the rheological properties rate of blood samples (https://doi.org/10.3390/MI14081594).

Niemitz et al. (https://doi.org/10.3390/MI14051062) developed a portable miniaturizable imaging system capable of rapid in vivo acquisition of images during diagnostic, surgical, or therapeutic procedures with a substantial reduction in artifacts due to specular reflections on glossy tissue surfaces. The two proposed strategies, i.e., the integration of cross-polarization using high-quality polarizers and multi-flash imaging for ex-post algorithmic reflection filtering, allow for the removal of image distortions, thus significantly improving the revelation of sub-surface tissue structures.

Gupta et al. (https://doi.org/10.3390/MI14071281) report the performance evaluation of an aperture-coupled terahertz antenna for microwave imaging of breast tissue. THz photons are sensitive to differences in water uptake between healthy and cancerous cells. The proposed sensor boasts a wide impedance bandwidth that allows radiation to penetrate tissue at varying depths, while providing high gain and excellent sensitivity to abnormal tissue.

A comparative study between new and old generation metal–oxide–semiconductor field-effect transistors (MOSFETs) is provided by Bakhoum et al. (https://doi.org/10.3390/MI14061135), who introduce a novel type of MOSFET that utilizes nanoporous gold (NPG) as a gate electrode. NPG, known for its excellent catalytic activity, is employed in this design. The MOSFET sensor was validated towards glucose and carbon monoxide detection.

This Special Issue also includes four review papers addressing cutting-edge topics in the field of biosensing. Specifically, these reviews take up topics related to the development of novel biosensing platforms for detecting cancer, viral, bacterial, and other pathological markers.

In the paper by Thao et al. (https://doi.org/10.3390/MI14051017), the authors explore the field of antioxidant nanozymes. These synthetic nanomaterials mimic the catalytic activities of natural antioxidant enzymes, such as catalase and superoxide dismutase. What makes them particularly interesting is their stability, cost-effectiveness, and customizability. Factors such as the size, composition, and surface modifications can be manipulated to fine-tune these nanozymes for various applications. The paper discusses the mechanisms behind antioxidant nanozymes, their potential impact in biomedicine, drug delivery, and biomarkers detection, and how they bridge the gap between natural enzymes and innovative nanotechnology.

A more specific topic concerning cancer is addressed in the review by Goldstein et al. (https://doi.org/10.3390/MI14051035), which describes several modern techniques for the detection of circulating tumor cells (CTCs). This approach, also known as “liquid biopsy”, allows for the minimally invasive detection of tumors due to the revelation of the presence of free tumoral cells in the bloodstream.

The review by Gholami et al. (https://doi.org/10.3390/MI14061185) discloses the potential of 3D carbon-based materials (i.e., graphene oxide, reduced graphene oxide, carbon nanotubes, and other carbon nanoallotropes) in the development of novel efficient theranostic strategies for the rapid diagnosis and treatment of viral hepatitis. In this context, the review by Battisti et al. (https://doi.org/10.3390/MI14081522) reports on a specific group of graphene oxide-based biosensors that rely on the fluorescence quenching effect that GO exerts on several fluorescent dyes and that can be exploited for the design of novel switch-ON and switch-OFF fluorescence biosensing platforms for the detection of a plethora of biomolecules, especially pathological biomarkers and environmental contaminants.

In conclusion, biosensors are widespread powerful tools used in both medical and environmental contexts. They play a pivotal role in detecting biomarkers, pollutants, xenobiotics, contaminants, and other analytes in complex samples. Over the past two years, interest in biosensing technologies has surged, driven by the pandemic’s impact and global environmental concerns. Researchers from diverse fields—chemistry, biology, physics, engineering, computation, and medicine—collaborate to optimize biosensors; their efforts focus on improving selectivity, sensitivity, and miniaturization. In this multidisciplinary field, novel biosensors are designed, exploring innovative sensing elements, immobilization techniques, and detection strategies. Micro- and nano-conception of these devices is a key area of research. Biosensors hold promise for a healthier future, leading to advancements in medical diagnosis processes and in both human and environmental health monitoring, and this Special Issue collects some of the most recent contributions to the field. These papers collectively contribute to the advancement of biosensing technologies, spanning through biomedical diagnostics, smart agriculture and environmental protection. Researchers worldwide continue to explore innovative approaches, and this Special Issue will serve as a valuable resource in its field for the scientific community.
